# Comparison between talazoparib and conventional chemotherapy in the treatment of HER2-positive breast cancer patients: A retrospective study

**DOI:** 10.3389/fimmu.2022.901636

**Published:** 2022-08-15

**Authors:** Ning Wang, Xiaopeng Yu

**Affiliations:** ^1^ Thoracic Surgery Department, Shengjing Hospital Affiliated to China Medical University, Shenyang, China; ^2^ Breast Surgery Department, Shengjing Hospital Affiliated to China Medical University, Shenyang, China

**Keywords:** talazoparib, chemotherapy, Kaplan-Meier, retrospective, HER2

## Abstract

**Background:**

The benefits of talazoparib compared with conventional chemotherapy in HER2-negative advanced breast cancer (ABC) remain unclear.

**Methods:**

Patients older than 18 years, with a deleterious germline BRCA1/2 (gBRCA1/2) mutated, metastatic, or locally advanced and HER2-positive breast cancer were enrolled. Patient data including age, menostatus, tumor grade, pathologic tumor size, lymph node status, and whether they had received adjuvant radiation or chemotherapy was collected. The primary outcomes of the study were disease-free survival (DFS), which was defined as the time from randomization to death or recurrence due to any reason, and overall survival (OS), which was defined as the time from randomization to death due to any reason. P<0.05 was considered to be statistically significant.

**Results:**

A total of 136 patients were finally enrolled in the present retrospective study, including 62 patients in the talazoparib group (group A) and 74 in the conventional chemotherapy group (group B). After a median follow-up of 70.9 months [95% confidence interval (CI): 68.3–78.5], both DFS and OS did not differ significantly between the two groups (P=0.658 and P=0.690, respectively). The exploratory subgroup analyses further validated the robustness of the primary results across the subgroups.

**Conclusions:**

Talazoparib was not better than conventional chemotherapy in terms of DFS and OS for the treatment of gBRCA1/2 mutated HER2-positive breast cancer patients.

## Introduction

Talazoparib is an oral drug that can inhibit poly (adenosine diphosphate ribose) polymerase (PARP) catalytic activity, and acts by effectively capturing PARP at damaged deoxyribonucleic acid (DNA) sites (1, 2). Damaged DNA cannot be repaired by homologous recombination repair in cancer cells with mutated breast cancer susceptibility genes 1 or 2 (BRCA1/2) (3).

The benefit of talazoparib in BRCA1/2-mutated patients with human epidermal receptor-2 (HER2) negative advanced breast cancer (ABC) has been demonstrated in several phase I/II clinical trials (4–6). The efficacy and safety of talazoparib in germlineBRCA1/2 (gBRCA1/2) mutated patients with HER2-negative ABC was compared with conventional chemotherapy based on the physician’s choice in the EMBRACA trial. As a result, talazoparib was found to be associated with a longer progression-free survival (PFS) compared to conventional chemotherapy [hazard ratio (HR) =0.542; 95% confidence interval (CI): 0.413–0.711; P<0.0001]. Additionally, there is a previous large, multicenter trial that compared the prognosis of patients with HER2-low and HER2-zero breast cancer, which showed a superior efficacy of the former population than the latter (7). However, the efficacy and safety of talazoparib in patients with HER2-positive ABC remains unclear. There is also evidence from pre-clinical studies evaluating the efficacy of PARP inhibitors in trastuzumab sensitive or resistant HER-2 positive cell lines and xenografts independent of BRCA status. Therefore, we hypothesized that HER-2 amplified tumors with BRCA1/2 may continue to be susceptible to PARP inhibitors mediated synthetic lethality. And so we performed the present retrospective study to assess the potential of using talazoparib in HER2-positive patients.

## Methods

### Patient selection

This retrospective was reported according to the Strengthening the Reporting of Observational Studies in Epidemiology (STROBE) reporting guidelines (8). Patients treated in our hospital between January 2012 and December 2019 were screened, and those meeting the following criteria were enrolled: (I) aged older than 18 years; (II) with locally advanced or metastatic HER2-positive breast cancer; (III) central testing showing a deleterious gBRCA1/2 mutation; (IV) had previously received less than four cytotoxic regimens and an anthracycline, a taxane, or both; and (V) received talazoparib or conventional chemotherapy in our hospital. Based on the treatments they received, the enrolled patients were further divided into two groups: those who received talazoparib (group A), and those who received conventional chemotherapy (group B). Treatment was not stopped until the disease progressed or an unacceptable toxicity occurred. After stopping treatment, the survival status and follow-up anti-cancer treatment of the patients were followed up every 12 weeks.

### Data collection and outcome evaluation

Patient data including age, menostatus, tumor grade, pathologic tumor size, lymph node status, and whether they received adjuvant radiation or conventional chemotherapy were collected. The primary outcomes of the study included disease-free survival (DFS), which was defined as the time from randomization to recurrence or death due to any cause, and overall survival (OS), which was defined as the time from randomization to death due to any reason. The OS of patients who were still alive at the time of data truncation or were lost during follow-up was recorded as the last known survival date of the patient. The treatment efficacy was investigated by performing exploratory subgroup analyses based on different baseline characteristics and demographic factors.

### Statistical analyses

Mann-Whitney tests or *t*-tests were used to compare continuous variables, while categorical variables were compared using Fisher exact tests or the χ^2^ test. Kaplan-Meier curves were performed to estimate the DFS and OS rates. All statistical analyses were carried out using R statistics, version 4.1.1 (R Foundation for Statistical Computing), and P<0.05 was considered statistically significant.

## Results

### Patients

**Figure 1:**
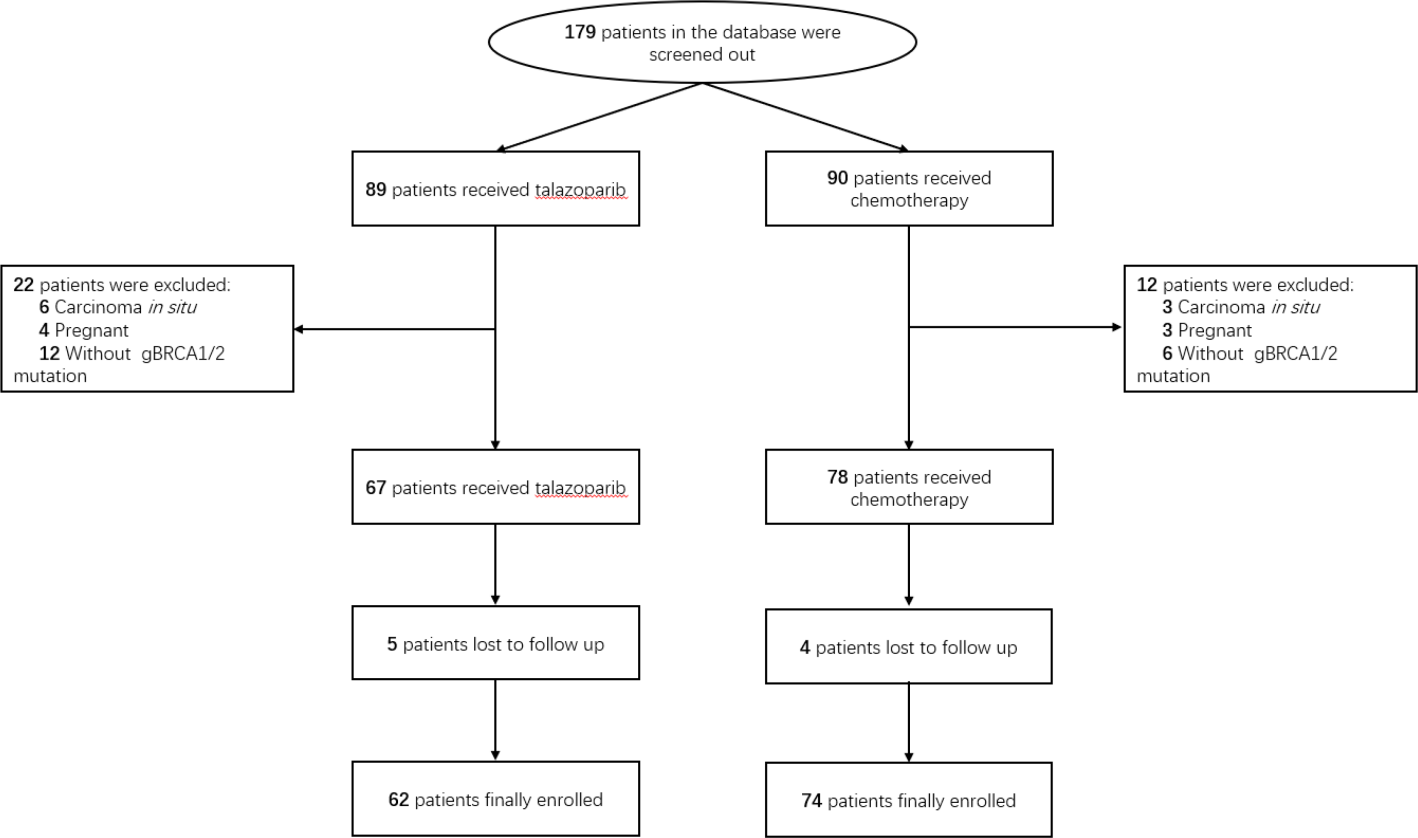
Patient selection process.

A total of 179 female patients [median age, 51 (IQR, 43–59) years] with deleterious gBRCA1/2 mutated HER2-positive ABC were screened from our hospital database. Among them, 89 patients received talazoparib and 90 patients received a physician’s choice of conventional chemotherapy. After searching the database, 34 patients were excluded for various reasons. Finally, we included 136 patients in the present retrospective study: 62 in the talazoparib group (group A) and 74 in the conventional chemotherapy group (group B), and the detailed flow diagram is displayed in **Figure 1**. As shown in **Table 1**, the baseline clinical characteristics, pathologic features, and staging were balanced between the two groups.

**Table 1:** Baseline characteristics of patients enrolled in the present study.

Characteristics	Total (N=136)	Talazoparib (N=62)	Chemotherapy (N=74)	*p* value
**Age, median [IQR], years**	51 [43–59]	50 [43–57]	51 [43–60]	0.66
**Menostatus, n (%)**				0.36
**Pre/peri**	76 (55.9)	32 (51.6)	44 (59.5)	
**Post**	60 (44.1)	30 (48.4)	30 (40.5)	
**Pathological tumor size, n (%)**				0.36
**≤2 cm**	91 (66.9)	39 (62.9)	52 (70.3)	
**>2 cm**	45 (33.1)	23 (37.1)	22 (29.7)	
**Stage**				0.66
**Locally advanced**	115 (84.6)	51 (82.3)	64 (86.5)	
**Metastatic**	21 (15.4)	11 (17.7)	10 (13.5)	
**Lymph node status, n (%)**				0.05
**Negative**	54 (39.7)	19 (30.6)	35 (47.3)	
**Positive**	82 (60.3)	43 (69.4)	39 (52.7)	
**Grade, n (%)**				
**I/II**	59 (43.4)	27 (43.5)	32 (43.2)	0.97
**III**	77 (56.6)	35 (56.5)	42 (56.8)	
**Adjuvant chemotherapy, n (%)**				0.47
**No**	109 (80.1)	48 (77.4)	61 (82.4)	
**Yes**	27 (19.9)	14 (22.6)	13 (17.6)	
**Adjuvant radiation, n (%)**				0.69
**No**	48 (35.3)	23 (37.1)	25 (33.8)	
**Yes**	88 (64.7)	39 (62.9)	49 (66.2)	

### Survival outcomes

**Figure 2:**
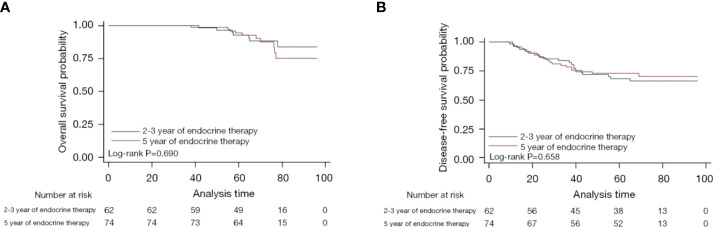
Kaplan-Meier curves for overall survival **(A)** and disease-free survival **(B)** of patients who received talazoparib versus chemotherapy.

After a median follow-up of 70.9 months (95% CI: 68.3–78.5), the DFS of patients who received talazoparib was estimated to be 68.4% (95% CI: 55.0–78.6%), while the OS of these patients was estimated to be 92.8% (95% CI: 81.9–97.2%). The DFS of patients who received conventional chemotherapy was estimated to be 73.0% (95% CI: 61.3–81.6%), while the OS of these patients was estimated to be 94.4% (95% CI: 85.7–97.9%). **Figure 2** shows the Kaplan-Meier curves of both DFS and OS between the two groups of patients, and it is clear that both DFS and OS did not differ significantly between the two groups (P=0.658 and P=0.690, respectively).

### Exploratory subgroup analyses

**Figure 3:**
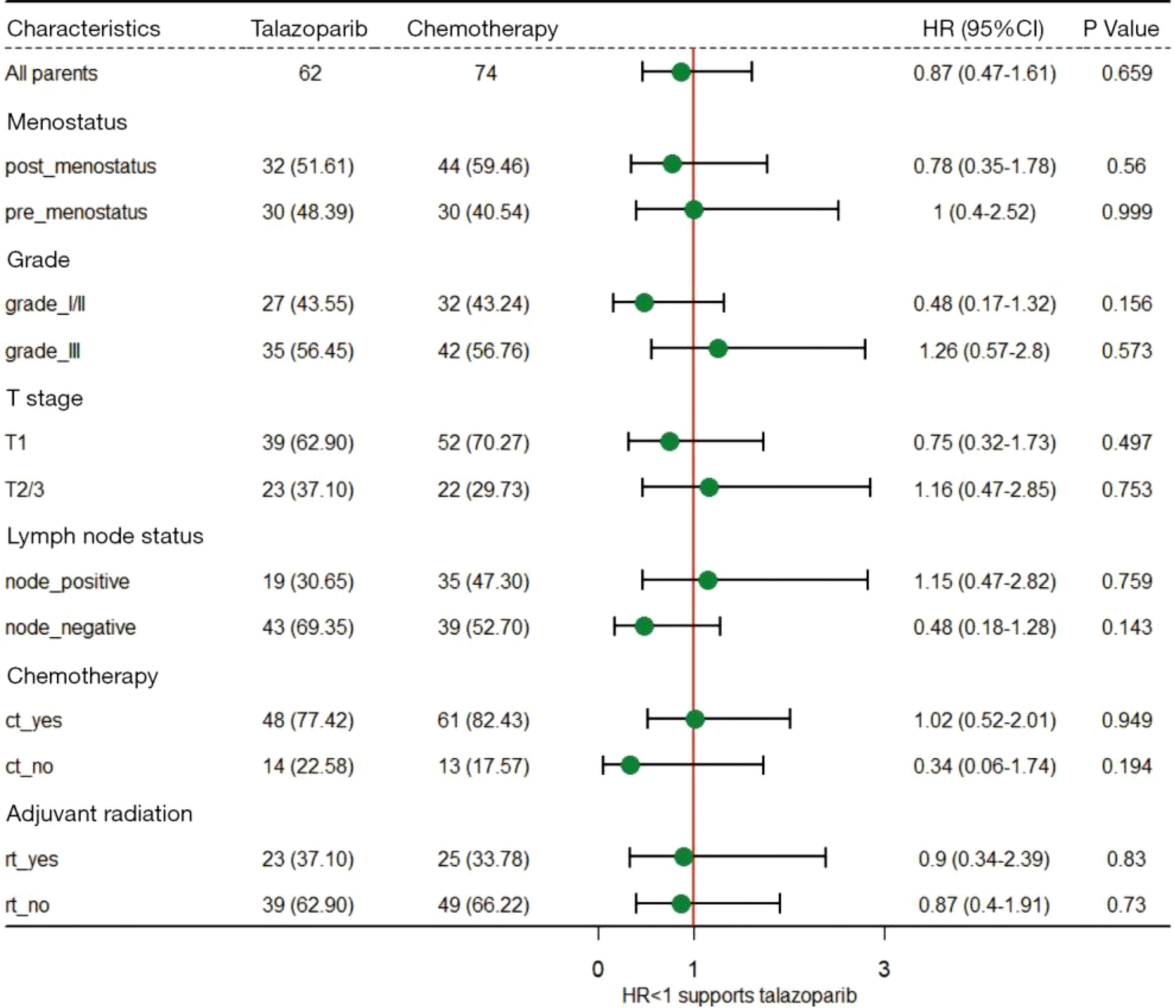
Exploratory subgroup analyses for disease-free survival.

**Figure 3** shows the results of the exploratory subgroup analyses. Subgroup analyses were performed based on different baseline characteristics. We found that none of the studied baseline factors altered the results, which further validated the robustness of our primary findings.

## Discussion

The results of this study, which compared the efficacy of talazoparib with conventional chemotherapy in the treatment of gBRCA1/2-mutated ABC patients, showed that there were no statistically significant differences in either DFS or OS between the two groups. Exploring the impact of follow-up treatment on progression after long-term survival post-progression (SPP; time from progression to death) is essential to understand the treatment effects evaluated in the trial. For cancers with longer SPPs, the variability of SPPs will be affected by subsequent treatment, thus diluting the OS benefits and minimizing the ability to detect statistical significance (9).

A previous randomized phase III trial comparing the efficacy of olaparib versus conventional chemotherapy (OlympiAD) (10) found that a statistically superior progression-free survival did not translate into a significantly better OS. Compared with the EMBRACA Trial (11, 12), the OlympiAD trial did not identify a statistically difference between the groups. A potential explanation was that the proportion of patients receiving follow-up PARP inhibitor treatment in the control group was lower in the OlympiAD trial than in the EMBRACA trial (8% *vs*. 33%). Since OlympiAD is the first phase III PARP trial, the availability of subsequent PARP inhibitors was limited. In both of the aforementioned trials, more than 40% of patients received follow-up platinum therapy (10). *BRCA1/2* reverse mutations that restore DNA repair capacity have been shown to lead to resistance to PARP inhibitors and platinum; therefore, the possible effect of subsequent platinum treatment deserves attention. Of note, a recent review also demonstrated a superior efficacy of PARP inhibitors than conventional conventional chemotherapy (6). The inconsistent result observed in our study should be further investigated. There are other differences between the results of these two phase III trials, but due to the differences in study design, patient characteristics, as well as the type and effect of follow-up treatment in the conventional chemotherapy group, cross-over trial comparison should be carried out cautiously. It is well known that chemotherapy regimens and chemotherapy cycles, adjuvant chemotherapy and chemotherapy cycles have an important impact on the prognosis of breast cancer patients, however, due to the limited data, we were unable to analyze these factors. Furthermore, this is a single center retrospective study, so the sample is not so representative. These limitations of our study should be noted.

In conclusion, neither DFS nor OS was significantly improved with talazoparib compared to conventional chemotherapy. Further exploratory subgroup analyses validated the robustness of our results across the subgroups. However, due to the small sample size and the retrospective design of our study, our results should be interpreted with caution, and further large-scale randomized studies are still urgently needed.

## Data availability statement

The original contributions presented in the study are included in the article/supplementary material. Further inquiries can be directed to the corresponding author.

## Ethics statement

This study was reviewed and approved by Shengjing Hospital Affiliated to China Medical University. Written informed consent was obtained from all participants for their participation in this study.

## Author contributions

(I) Conception and design: NW and XY; (II) administrative support: NW and XY; (III) provision of study materials or patients: NW and XY; (IV) collection and assembly of data: NW and XY; (V) data analysis and interpretation: NW and XY; (VI) manuscript writing: all authors. All authors contributed to the article and approved the submitted version.

## Conflict of interest

All authors have completed the ICMJE uniform disclosure form.

## Publisher’s note

All claims expressed in this article are solely those of the authors and do not necessarily represent those of their affiliated organizations, or those of the publisher, the editors and the reviewers. Any product that may be evaluated in this article, or claim that may be made by its manufacturer, is not guaranteed or endorsed by the publisher.

## References

[1] Wang B Chu D Feng Y. Discovery and characterization of (8S,9R)-5-Fluoro-8-(4-fluorophenyl)-9-(1-methyl-1H-1,2,4-triazol-5-yl)-2,7,8,9-tetrahydro-3H-pyrido[4,3,2-de]phthalazin-3-one (BMN 673, talazoparib), a novel, highly potent, and orally efficacious Poly(ADP-ribose) polymerase-1/2 inhibitor, as an anticancer agent. J Med Chem (2016) 59:335–57. doi: 10.1021/acs.jmedchem.5b01498 26652717

[2] Murai J Huang SY Renaud A. Stereospecific PARP trapping by BMN 673 and comparison with olaparib and rucaparib. Mol Cancer Ther (2014) 13:433–43. doi: 10.1158/1535-7163.MCT-13-0803 PMC3946062 24356813

[3] Lord CJ Ashworth A. PARP inhibitors: Synthetic lethality in the clinic. Science (2017) 355:1152–8. doi: 10.1126/science.aam7344 PMC6175050 28302823

[4] de Bono J Ramanathan RK Mina L Phase I. Dose-escalation, two-part trial of the PARP inhibitor talazoparib in patients with advanced germline BRCA1/2 mutations and selected sporadic cancers. Cancer Discovery (2017) 7:620–9. doi: 10.1158/2159-8290.CD-16-1250 PMC5905335 28242752

[5] Turner NC Telli ML Rugo HS. A phase II study of talazoparib after platinum or cytotoxic nonplatinum regimens in patients with advanced breast cancer and germline BRCA1/2 mutations (ABRAZO). Clin Cancer Res (2019) 25:2717–24. doi: 10.1158/1078-0432.CCR-18-1891 30563931

[6] Ricci AD Rizzo A Rojas Llimpe FL. Novel HER2-directed treatments in advanced gastric carcinoma: AnotHER paradigm shift? Cancers (Basel) (2021) 13. doi: 10.3390/cancers13071664 PMC8036476 33916206

[7] Tan R Ong WS Lee KH. HER2 expression, copy number variation and survival outcomes in HER2-low non-metastatic breast cancer: an international multicentre cohort study and TCGA-METABRIC analysis. BMC Med (2022) 20:105. doi: 10.1186/s12916-022-02284-6 35296300 PMC8928638

[8] von Elm E Altman DG Egger M. The strengthening the reporting of observational studies in epidemiology (STROBE) statement: guidelines for reporting observational studies. J Clin Epidemiol (2008) 61:344–9. doi: 10.1016/j.jclinepi.2007.11.008 18313558

[9] Broglio KR Berry DA. Detecting an overall survival benefit that is derived from progression-free survival. J Natl Cancer Inst (2009) 101:1642–9. doi: 10.1093/jnci/djp369 PMC4137232 19903805

[10] Robson ME Tung N Conte P. OlympiAD final overall survival and tolerability results: Olaparib versus chemotherapy treatment of physician's choice in patients with a germline BRCA mutation and HER2-negative metastatic breast cancer. Ann Oncol (2019) 30:558–66. doi: 10.1093/annonc/mdz012 PMC6503629 30689707

[11] Litton JK Rugo HS Ettl J. Talazoparib in patients with advanced breast cancer and a germline BRCA mutation. N Engl J Med (2018) 379:753–63. doi: 10.1056/NEJMoa1802905 PMC10600918 30110579

[12] Ettl J Quek RGW Lee KH. Quality of life with talazoparib versus physician's choice of chemotherapy in patients with advanced breast cancer and germline BRCA1/2 mutation: patient-reported outcomes from the EMBRACA phase III trial. Ann Oncol (2018) 29:1939–47. doi: 10.1093/annonc/mdy257 30124753

